# Human behavior in image-based Road Health Inspection Systems despite the emerging AutoML

**DOI:** 10.1186/s40537-022-00646-8

**Published:** 2022-07-20

**Authors:** Thitirat Siriborvornratanakul

**Affiliations:** Graduate School of Applied Statistics, National Institute of Development Administration (NIDA), 148 SeriThai Rd., Bangkapi, Bangkok, 10240 Thailand

**Keywords:** Human Behavior, AutoML, Automated Machine Learning, Artificial Intelligence, Machine Learning, Road Health Inspection

## Abstract

**Introduction:**

The emergence of automated machine learning or AutoML has raised an interesting trend of no-code and low-code machine learning where most tasks in the machine learning pipeline can possibly be automated without support from human data scientists. While it sounds reasonable that we should leave repetitive trial-and-error tasks of designing complex network architectures and tuning a lot of hyperparameters to AutoML, leading research using AutoML is still scarce. Thereby, the overall purpose of this case study is to investigate the gap between current AutoML frameworks and practical machine learning development.

**Case description:**

First, this paper confirms the increasing trend of AutoML via an indirect indicator of the numbers of search results in Google trend, IEEE Xplore, and ACM Digital Library during 2012–2021. Then, the three most popular AutoML frameworks (i.e., Auto-Sklearn, AutoKeras, and Google Cloud AutoML) are inspected as AutoML’s representatives; the inspection includes six comparative aspects. Based on the features available in the three AutoML frameworks investigated, our case study continues to observe recent machine learning research regarding the background of image-based machine learning. This is because the field of computer vision spans several levels of machine learning from basic to advanced and it has been one of the most popular fields in studying machine learning and artificial intelligence lately. Our study is specific to the context of image-based road health inspection systems as it has a long history in computer vision, allowing us to observe solution transitions from past to present.

**Discussion and evaluation:**

After confirming the rising numbers of AutoML search results in the three search engines, our study regarding the three AutoML representatives further reveals that there are many features that can be used to automate the development pipeline of image-based road health inspection systems. Nevertheless, we find that recent works in image-based road health inspection have not used any form of AutoML in their works. Digging into these recent works, there are two main problems that best conclude why most researchers do not use AutoML in their image-based road health inspection systems yet. Firstly, it is because AutoML’s trial-and-error decision involves much extra computation compared to human-guided decisions. Secondly, using AutoML adds another layer of non-interpretability to a model. As these two problems are the major pain points in modern neural networks and deep learning, they may require years to resolve, delaying the mass adoption of AutoML in image-based road health inspection systems.

**Conclusions:**

In conclusion, although AutoML’s utilization is not mainstream at this moment, we believe that the trend of AutoML will continue to grow. This is because there exists a demand for AutoML currently, and in the future, more demand for no-code or low-code machine learning development alternatives will grow together with the expansion of machine learning solutions. Nevertheless, this case study focuses on selected papers whose authors are researchers who can publish their works in academic conferences and journals. In the future, the study should continue to include observing novice users, non-programmer users, and machine learning practitioners in order to discover more insights from non-research perspectives.

## Introduction

### Background and motivation

**Fig. 1:**
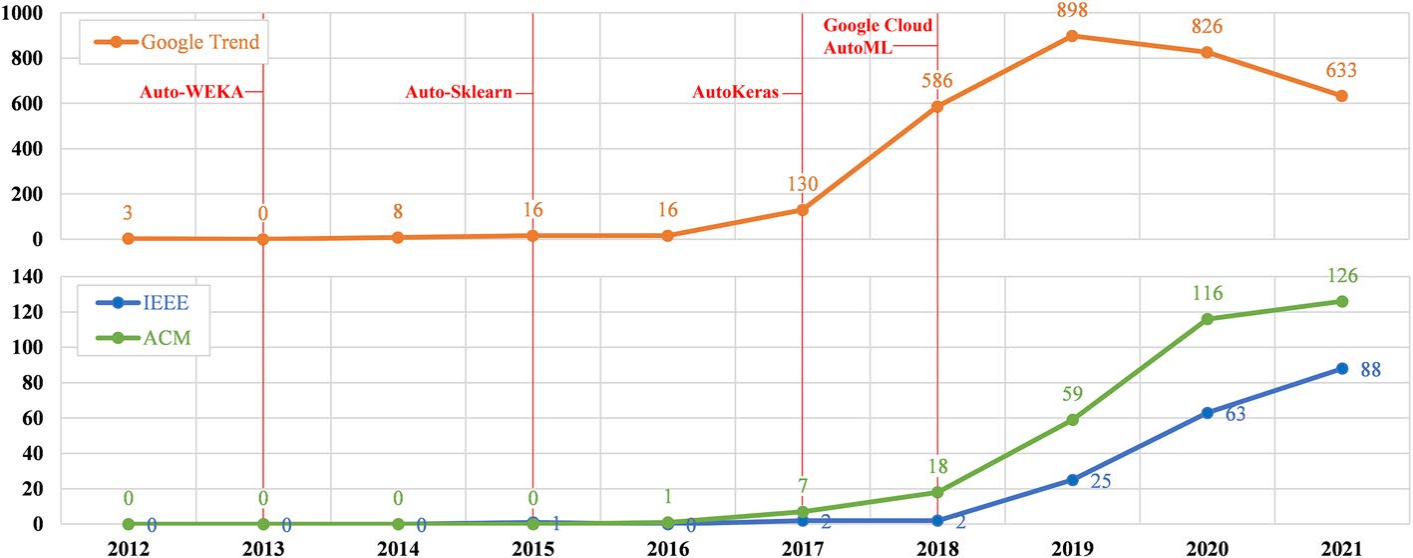
The rising trend of AutoML in Google Trend (worldwide), IEEE Xplore, and ACM Digital Library during 2012 to 2021. The vertical axis represents the numbers of search results according to the “AutoML” search keyword in each year. Note that values plotted in this figure were manually collected from each search engine on December 15, 2021, without any coding or scraping software involved

In this era of the artificial intelligent boom, many people have high expectations that artificial intelligence is a magic tool that can automate any task and solve any problem. Among several artificial intelligent techniques, machine learning has significantly dominated the field lately up to the point that resulted in 12x growth in business hiring according to LinkedIn 2018 as mentioned in [14]. Driven by the rapid growth of machine learning adoption, one of the most mentioned artificial intelligent tools is automated machine learning or AutoML—a machine learning tool or framework that is, in its ideal form, capable of automating the entire machine learning pipeline. Figure 1 illustrates the worldwide rising trend of AutoML regarding Google Trend and two prestigious academic databases (i.e., IEEE and ACM); note that we do not include the search keyword of “automated machine learning” when creating this figure, as many works refer to this term as an automatic system involving one or more machine learning algorithms, not AutoML as intended by this paper. This rising trend in AutoML has raised an interesting question regarding human factors in machine learning development—is it still necessary to have human data scientists designing and developing a machine learning system?

Nevertheless, according to the survey of AutoML opportunities for healthcare by Waring et al. in 2020 [30], there were few works that applied AutoML to healthcare regardless of demonstrated need. This is similar to the 2020 survey of AutoML conducted by Kaggle [21] on 20,036 respondents. In this survey, 13,341 respondents (66.6%) did not answer to the AutoML question; 4679 respondents (23.4%) did not use AutoML; 2016 respondents (10.1%) used some form of AutoML. In other words, 69.9% of Kaggle respondents that answered the AutoML question said that they did not use any AutoML at all.

The contradiction between the rising trend of AutoML and the small number of actual usages even in the major machine learning community has inspired this paper. Finding the cause(s) of this contradiction is neither easy nor straightforward. This is because of the huge possibility of recent machine learning solutions that have been rooted in various data types and have been branched out into many applications which can be completely different from one another in terms of domain knowledge, problem formulation, type of models, interpretation, evaluation, etc.

### Research methodology

In this paper, we choose image-based research as our case study because of three main reasons. (1) Computer vision is one of the most popular research fields lately. According to the numbers of articles published in arXiv shown in Table 1, machine learning and computer vision are the top two most popular sub-categories among arXiv’s 40 computer-science sub-categories. (2) Computer vision has a longer history (compared to AutoML) and involves high diversity problems, spanning across artificial intelligence as well as machine learning techniques of various difficulty levels from basic to advanced. Hence, studying AutoML in computer vision allows investigation of how and why solutions have evolved from traditional rule-based methods to modern machine learning methods, and finally to AutoML methods. (3) Lately, computer vision researchers and practitioners have significantly relied on using a machine learning backbone to yield practical results in mass adoption; machine learning (i.e., deep learning to be more specific) has become indispensable in computer vision communities. Thereby, any conclusion drawn from the field of computer vision should mean a lot in the future development of machine learning.

**Table 1:** The top ten most popular computer science’s sub-categories ranked by the numbers of arXiv’s search results (the first column) from highest to lowest

N	Category	
	Code	Detail
108,709	cs.LG	Machine learning
74,677	cs.CV	Computer vision and pattern recognition
46,390	cs.AI	Artificial intelligence
38,974	cs.IT	Information theory
35,122	cs.CL	Computation and language
22,034	cs.CR	Cryptography and security
19,403	cs.DS	Data structures and algorithms
18,573	cs.RO	Robotics
17,775	cs.NI	Networking and internet architecture
16,121	cs.DC	Distributed, parallel, and cluster computing

Note that the numbers shown in this table were manually collected by searching each category code (the second column) in https://arxiv.org/search/ on May 9, 2022, without any coding or scraping software involved

Because of these reasons, we believe that computer vision is a good starting point for our AutoML case study. However, as studying every aspect of computer vision is too complicated and may confuse readers, our investigation chooses to focus on the context of image-based road health inspection as it is one of the long-standing problems in computer vision for civil engineering. By exploring research papers on image-based road health inspection, our goal is twofold—(1) to clarify which human-related tasks should better be replaced by AutoML and which ones cannot be easily replaced by AutoML at this moment; (2) to imply the readiness of AutoML frameworks for mass adoption (at the time of writing this paper).

**Fig. 2:**
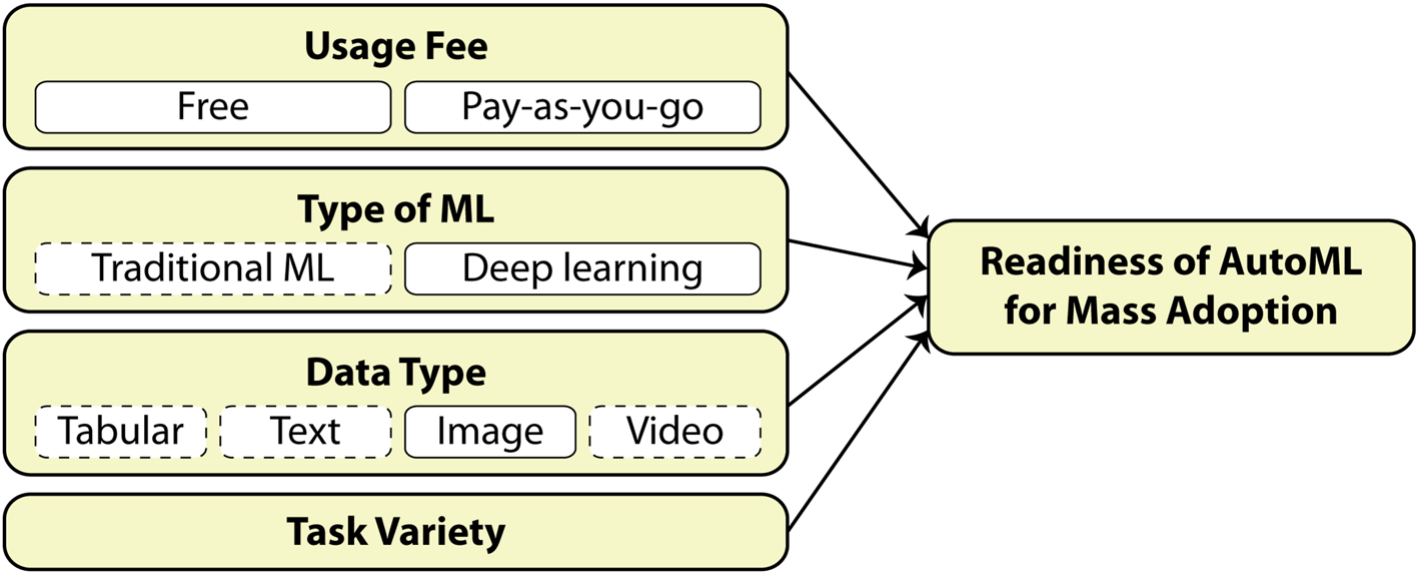
The conceptual framework

To achieve the aforementioned goal, the study of this paper is designed to be a qualitative exploratory research where only some representative works are closely explored instead of exploring a lot of works to yield some statistically significant results. Figure 2 shows our conceptual framework that implies the readiness of AutoML for mass adoption regarding computer vision practitioners, despite the rising trend of no-code and low-code machine learning as demanded by non-technical users and machine learning beginners. This implication is drawn by observing four related factors (i.e., usage fee, machine learning type, data type, and task variety) regarding the three selected representative AutoML frameworks (more detail about these selected frameworks in “AutoML’s background” section). Note that because recent works in image-based road health inspection have been dominated significantly by deep learning, most of the representative works selected for our discussion (in “AutoML in image-based road health inspection systems” section) rely on deep learning not traditional machine learning; omitting the factor of “Traditional ML” from this study is illustrated by the dashed line box in Fig. 2. The same as non-image data types which are not discussed in our selected works.

### Related works and research contributions

AutoML is an emerging trend and still an active research area whose increasing demand has been driven by the recent growth in machine learning both academically and commercially. Therefore, the number of works in AutoML is still relatively small compared to other mature study fields.

In 2020, Waring et al. [30] conducted a survey on AutoML for healthcare, including both traditional machine learning and deep learning. The authors walked through and discussed each technical step in the machine learning pipeline that could be automated by AutoML, focusing on open-source AutoML frameworks like Auto-WEKA, Auto-Sklearn, TPOT, etc. In the end, they concluded opportunities as well as limitations of AutoML in biomedical environments. In short, although the need existed, few works were done to apply AutoML in healthcare. Key challenges of deploying AutoML from a healthcare perspective include the lack of high-quality data, the lack of decision transparency, and AutoML’s inefficiency for large datasets that are usually found in biomedical environments.

In 2021, Zoller and Huber [33] claimed their work to be the largest and most extensive evaluation of AutoML frameworks as they evaluated six different AutoML frameworks (i.e., TPOT, Hyperopt-Sklearn, Auto-Sklearn, Random Search, ATM, and H2O) on 73 real datasets. However, they only focused on traditional machine learning; Neural Architecture Search (NAS), a popular sub-topic of AutoML, was unrelated and therefore excluded. The authors concluded that all experimental AutoML frameworks performed similarly on average and, somehow, some datasets were better suited for AutoML than others. Many limitations of AutoML were discussed, particularly the fact that most AutoML frameworks focused on automating a single point of the machine learning pipeline whereas no work did automate the most time-consuming step of data cleaning. Besides, for truly novice users, AutoML frameworks still provided no support in automating data acquisition and deployment measures.

Another AutoML survey in 2021 was proposed by He et al. [12]. Like Waring et al. [30] described earlier, this paper walked through each technical step in the machine learning pipeline (i.e., data preparation, feature engineering, model generation, and model evaluation) and discussed the recent progress of corresponding AutoML techniques. Not putting their main focus on the complete AutoML frameworks, this paper made extensive NAS performance comparison based on technical evaluation metrics (i.e., the number of parameters, top-1 accuracy, top-5 accuracy, and GPU days) regarding different NAS techniques proposed during 2017–2020 in prestigious academic conferences; the comparison was based on the task of image classification regarding two image datasets (i.e., CIFAR-10 and ImageNet). In the end, the authors pointed out that early NAS studies focused on high performance despite the resource consumption that could go extremely high like the 3150 GPU days of Google AmoebaNet (AAAI 2019) [9]. However, recent NAS studies tried to compromise both the search efficiency and the performance.

Up to this point, it can be seen that the main focus of AutoML research papers is often on the technical and algorithmic aspects regarding some popular steps of the machine learning pipeline like NAS and hyperparameter optimization. However, there are other research papers that explore AutoML from another perspective. For example, the work of Drozdal et al. [4] in 2020 conducted three experiments to reveal which information impacted human data scientists to trust the models produced by AutoML. The authors concluded that transparency and understandability in AutoML help increase user trust. Besides, when the trust was established, they found that the top two AutoML features that were important to human data scientists were model performance metrics and visualizations. In 2021, Mohr et al. [20] studied the ineffectiveness of AutoML process that often ended up being canceled due to a timeout. The authors proposed a new solution to predict the run time of AutoML, increasing AutoML successful evaluations and improving AutoML best solutions. The latest AutoML survey of Karmaker et al. [14] in 2022 proposed a new system that classifies several AutoML systems and frameworks based on their level of autonomy, automated tasks, the ability of domain experts to access machine learning, and the efficiency of human data scientists. The lowest level (Level 0) means the whole process is entirely manual whereas the highest level (Level 6) means everything is entirely automated, including the steps of task formulation, prediction engineering, and result summarizing and recommendation.

From these existing surveys in AutoML, it seems like, at this particular moment, there is no tool or framework yet that is capable of automating every single step in the machine learning pipeline. Nevertheless, there is the work of Zheng et al. [32] in 2021 that proposed a new paradigm of the fully AutoML pipeline. Proving the potential of this paradigm by a novel evolutionary algorithm with lifelong knowledge anchors, the authors were able to win the AutoDL 2019 challenge^1^ [17] and yielded the state-of-the-art performance on many datasets and modalities (i.e., image, video, text, speech, and tabular). The final goal of this work is to develop a future AutoML framework that can automate not only model selection/generation/training but also data preprocessing, feature engineering, and ensembling of an arbitrary dataset as well as evaluation metric.

After reviewing works that survey recent AutoML frameworks and concerns, we found that most previous works focus on comparing the technical and algorithmic aspects of AutoML which are unnecessarily complicated for most machine learning practitioners. The exception is the work of Drozdal et al. [4] which studies the trust of human data scientists toward AutoML. In the part of judging AutoML’s readiness for practical uses, there is only the work of Waring et al. [30] that aligns the investigation of AutoML with actual healthcare usage scenarios, allowing readers to judge AutoML’s readiness in the context of healthcare. Nevertheless, to the best of our knowledge, there is no previous work that studies the readiness of AutoML for practical uses based on the pure perspective of machine learning practitioners, particularly in the image-based deep learning context where image classification is not the only task. In conclusion, our contributions are threefold:

1. We propose a new case study research where the readiness of AutoML is closely investigated from the perspective of machine learning practitioners. This includes choosing three representative AutoML frameworks which are the most popular according to practitioners’ vote, and providing investigation factors (as shown in Fig. 2) that are easy to understand and follow by practitioners.
2. Unlike previous works, our investigation factor includes many image-based tasks that are beyond image classification.
3. We draw a conclusion that is similar to other previous works—current AutoML frameworks are not ready yet for computer vision practitioners, despite the rising demand from novice users and machine learning practitioners. However, the key challenges we discover (in “Discussion and evaluation” section) are different from previous works [4, 12, 14, 20, 30, 33] as ours are specifically drawn from the context of image-based road health inspection that, to the best of our knowledge, has never been studied elsewhere.

For the rest of this paper, we will first explain the background of AutoML and its promised capabilities in “AutoML’s background” section, focusing on popular AutoML frameworks among practitioners. Then in “AutoML in image-based road health inspection systems, we will dig into recently published works (including ours) of automatic image-based road health inspection systems, conclude human-related tasks in the development of these systems, and analyze why these recent works did or did not utilize AutoML. “Discussion and evaluation” section will discuss results from our exploratory study. Finally, “Conclusions” section will conclude this case study paper.

## Case description

### AutoML’s background

Speaking of the history of machine learning, it was back in 1943 when McCulloch and Pitts [19] proposed the first mathematical model of neural networks. Then in 1955, the term “artificial intelligence” was invented in a proposal by McCarthy et al. Few years later in 1959, the term “machine learning” was coined and popularized by Samuel [24]. Despite this long history of machine learning, it was not until 2013 that the very first AutoML framework named Auto-WEKA [8] was proposed by the University of British Columbia, Canada. According to [8], Auto-WEKA was a freely downloadable software that simultaneously selected classification algorithms and performed hyperparameter optimization. This fully automated framework utilized Bayesian optimization to choose among 39 classification algorithms (27 base classifiers, 10 meta methods, and 2 ensemble methods), 3 feature search methods, and 8 feature evaluators. Experiments were conducted on 21 datasets including some image datasets like MNIST and CIFAR-10. The authors concluded that Auto-WEKA often outperformed other algorithm selection and hyperparameter optimization methods, particularly on large datasets.

Following Auto-WEKA, other alternative AutoML frameworks have been proposed continuously. According to the 2020 survey of Kaggle [21], the most popular usages of AutoML were ranked as automated model selection (40.4%), automated hyperparameter tuning (33.8%), automated data augmentation (32.4%), automation of full machine learning pipelines (28.9%), automated feature engineering (26.1%), auto-model architecture searches (11.1%), and others (6.5%). This Kaggle survey also concluded that the top three AutoML frameworks were Auto-Sklearn [7] (29.1%), AutoKeras [13] (20.9%), and Google Cloud AutoML^2^ (18.1%) respectively. Figure 1 marks these top three frameworks and Auto-WEKA for better visualization of the rising AutoML trend in correlation to the introduction of popular AutoML frameworks. From the figure, it can be seen that Google Cloud AutoML was introduced at an exact moment when the rising trend in AutoML was quite obvious.

For the rest of this section, we will explore these top three AutoML frameworks in detail and compare them from machine learning practitioners’ perspectives. Our focused aspects include (1) usage license and fee, (2) programming language and library dependency, (3) coverage of machine learning types (i.e., traditional machine learning, deep learning, or both), (4) computational units like central processing unit (CPU), graphics processing unit (GPU), and tensor processing unit (TPU), (5) supporting tasks, and (6) supporting data types (e.g., tables, images, texts, etc.).

#### Auto-Sklearn (2015)

Starting from the most popular framework named Auto-Sklearn [7] that was first introduced in 2015 and has been said to be a by-product of the ChaLearn AutoML Challenge competitions^3^ (2015–2018) [10]. Auto-Sklearn is a free and open-source Python library with the 3-clause BSD license. At the time of writing this paper, Auto-Sklearn 0.14.2 requires a user to use at least Python 3.7. As its name suggests, Auto-Sklearn mainly relies on the popular Scikit-Learn machine learning library for data preparation, data transformation, and machine learning algorithms. Hence, any choice of data preparation/transformation and machine learning algorithms beyond Scikit-Learn is not available in Auto-Sklearn as well. As for computational units, basic parallel computation on CPU is provided in Auto-Sklearn. However, as both Scikit-Learn and Auto-Sklearn are not specifically designed for deep learning, there is no GPU and TPU support to help speed up the calculation.

In detail, Auto-Sklearn uses a Bayesian optimization search to find a top-performing model pipeline regarding a given input dataset. Apart from algorithm selection and hyperparameter tuning, Auto-Sklearn is also capable of automatically creating an ensemble of top-performing models as well as learning from models that perform well on similar datasets. Finally, there is no clear restriction about what types of inputs cannot be used in Auto-Sklearn. For structured data and tabular data, they are popular data types used in Scikit-Learn so there should not be problems in Auto-Sklearn. For natural language or text data, there are many non-deep learning tasks that can be performed by Scikit-Learn and Auto-Sklearn. For image data, although some tasks can be done in Scikit-Learn and Auto-Sklearn, Scikit-Learn is not a recommended library for image analytics and computer vision as it provides limited image-based solutions and has no hardware acceleration to help process a large image dataset.

#### AutoKeras (2017)

The next AutoML framework to be discussed is AutoKeras [13] which was first introduced in 2017. Like Auto-Sklearn, AutoKeras is a free and open-source Python library with the Apache -2.0 license. The word “Keras” refers to an open-source Python library (first released in 2015) that acts as a high-level interface for several deep learning backends (i.e., TensorFlow, Theano, and CNTK). Keras is popular for its ease of use which allows beginners and practitioners to rapidly prototype a deep learning model with a few lines of code. Since 2019, Google has included Keras as the official high-level API for TensorFlow 2 and it has remained like that ever since. At the time of writing this paper, AutoKeras 1.0.16 requires a user to have at least Python 3.5 and TensorFlow 2.3.0. Because AutoKeras currently relies on TensorFlow 2 Keras, the model provided by AutoKeras is a TensorFlow 2 Keras (tf.keras) model, not a standalone Keras model. Also, most capabilities of AutoKeras mainly depend on TensorFlow 2 Keras. This means that, like TensorFlow 2 Keras, AutoKeras is specially designed for neural networks and deep learning not traditional machine learning, and AutoKeras can be run in parallel on CPU, GPU, or TPU.

In detail, AutoKeras performs Neural Architecture Search (NAS) that tries to discover the best performing model architecture and its hyperparameters; this includes a new framework of Bayesian optimization for efficient search. AutoKeras supports several deep learning and neural network tasks on several input data (both structured and unstructured data). For example, image classification/regression, text classification/regression, structured data classification/regression. Tasks that AutoKeras hasn’t supported yet at the time of writing this paper, are time series forecasting, object detection, and image segmentation. For advanced users, AutoKeras allows customizing the search space with some high-level configurations.

#### Google Cloud AutoML (2018)

The last AutoML framework to be discussed in this paper is Google Cloud AutoML which was introduced in early 2018. Unlike Auto-Sklearn and AutoKeras, Google Cloud AutoML is an enterprise cloud solution that requires a credit card number upon registration. In return, a new customer is granted a free credit of US$ 300–400 at their first registration. As one of the world’s leading machine learning cloud providers, Google Cloud AutoML provides a unified API, client library, and user interface all in one place. Client API libraries are provided in several languages (e.g., Go, Java, Node.js, Python) depending on the choice of AutoML services. The whole machine learning pipeline, from the very first step of importing data to the very last steps of reporting the model’s performances in many evaluation metrics as well as exporting the model for deployment, is provided as a step-by-step GUI that barely requires any programming skill.

Speaking of computational units, parallel/distributed CPU, GPU, and TPU computations are all supported and more computational units are available upon request. Several AutoML services are available in Google Cloud AutoML, for example, AutoML Tables (for structured data), AutoML Vision (for image data), AutoML Video Intelligence (for video data), and AutoML Natural Language (for text data). Available tasks include classification, regression, object detection, object tracking, and action recognition. Of course, all these services come at a pay-as-you-go pricing strategy which depends on several criteria—for example, machine type, computational unit(s), usage time (per node hour), data type (image, video, text, or tabular), task (classification, object detection, action recognition, etc.), usage mode (training or prediction), etc. An extra fee is also charged for AutoML Edge as the model will get further optimized into a format that is suitable for being deployed on an edge device.

**Fig. 3:**
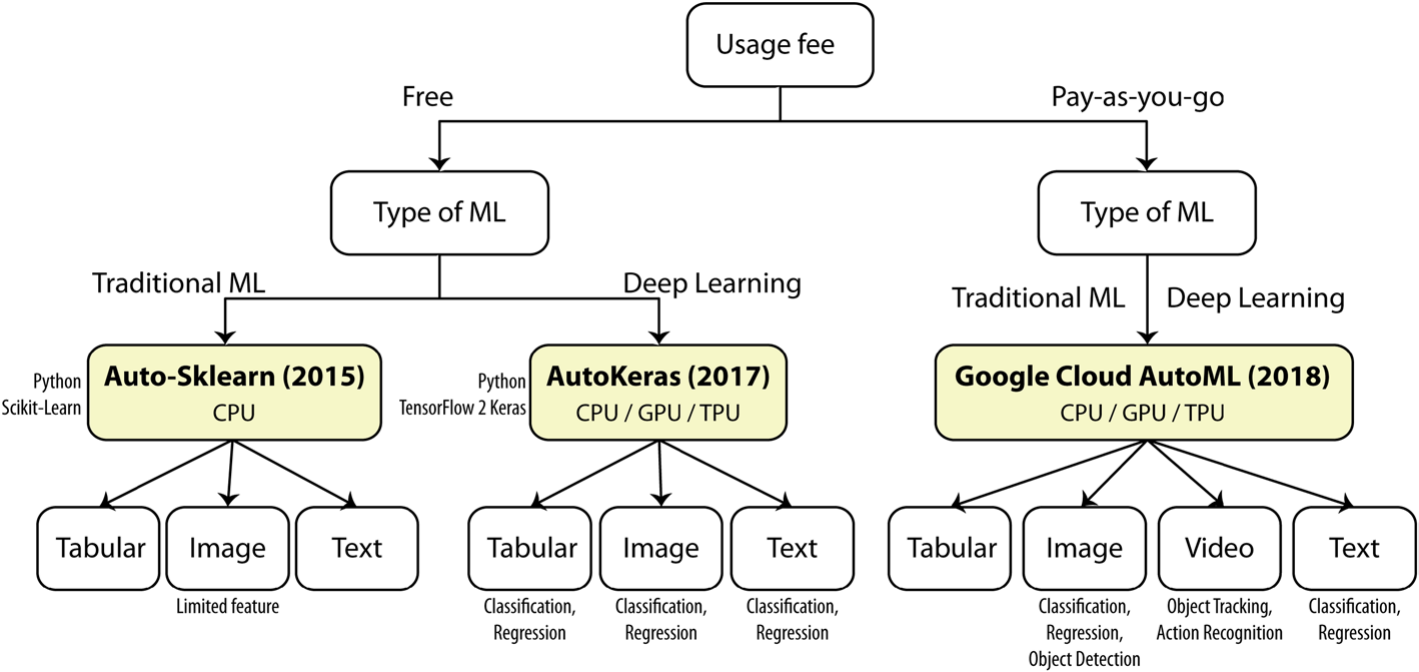
Our decision diagram regarding the three AutoML frameworks discussed in “AutoML’s background” section

To conclude this section, Fig. 3 shows our decision diagram to help practitioners choose among the three most popular AutoML frameworks. It can be seen that the top two most popular AutoML frameworks are free but highly dependent on specific Python libraries that require a user to possess some programming skill. Choosing between these two free frameworks is straightforward—Auto-Sklearn for traditional machine learning and AutoKeras for neural network and deep learning. The non-free Google Cloud AutoML provides a bundle of professional services that require less to no programming skill. Many services are included in Google Cloud AutoML so that it spans both traditional machine learning and deep learning techniques and supports many data types.

Nevertheless, most available AutoML frameworks have currently aimed for automated supervised learning and have not yet included ideas like self-supervised learning (SSL) and Automated Semi-Supervised Learning (AUTO-SSL) [16]. Also, it is obvious that using AutoML still cannot replace human tasks of gathering, mixing, cleaning, and preparing a good-quality input dataset; this concern is also mentioned in the 2021 survey of Zoller and Huber [33]. In addition, because of the nature of supervised learning, it still requires humans to think about how to reasonably and effectively divide a limited number of data into a set of appropriate output classes.

### AutoML in image-based road health inspection systems

**Fig. 4:**
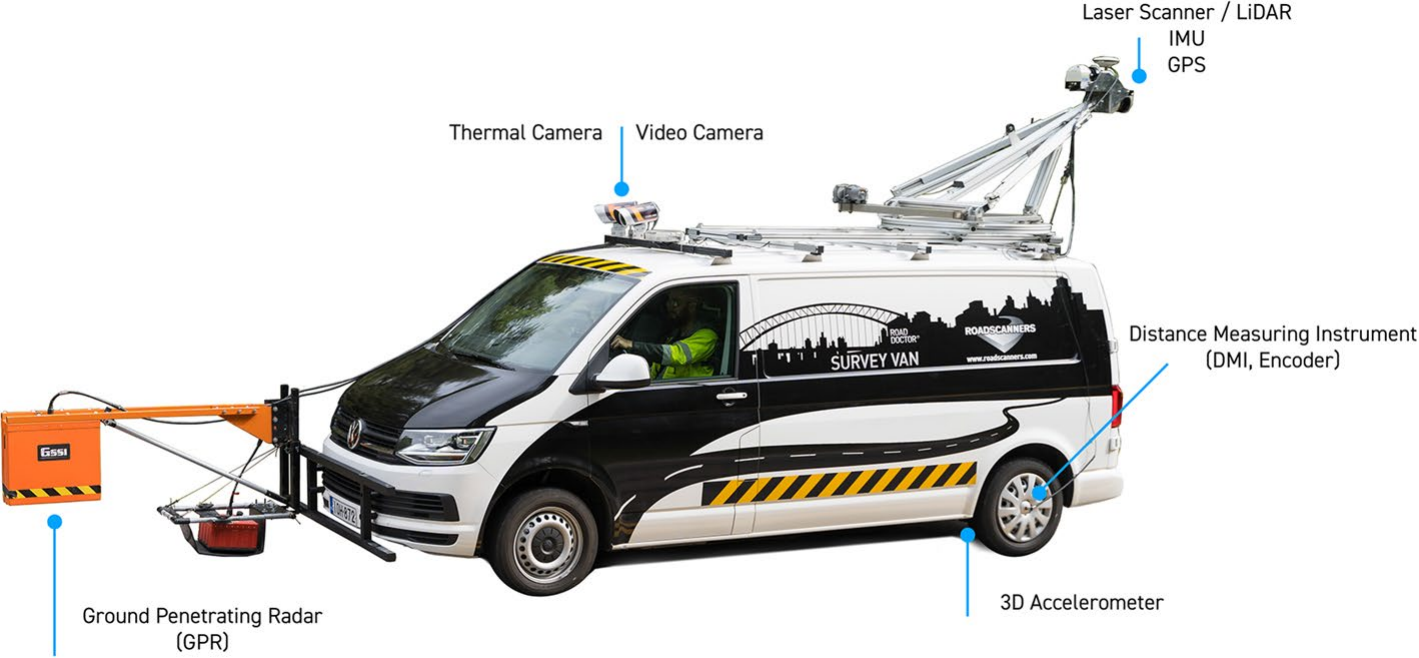
An example of a road scanning vehicle. This image is retrieved on May 15, 2022 from https://www.roadscanners.com/products/road-clinic-rdsv/full-rdsv-system-road-data-collection/

In order to portray AutoML usages in actual image-based machine learning development, this section explores a specific context of image-based road health inspection systems which is one of the long-standing concerns in civil engineering. Speaking of road health inspection systems, they often refer to automated systems which are designed for monitoring road surface conditions and recognizing any surface damage (if any) in order to fix them fast before the damage gets worse. A big road scanning vehicle, as an example shown in Fig. 4, is a common form of road health inspection system that has been owned by government sectors in many countries and is also available as a professional pay-per-use service. To detect and recognize road conditions as well as damages, road health inspection systems usually analyze and interpret data retrieved from one or more sensors. There are many kinds of sensors that can be used together to form a smart road health inspection system. One of the popular sensors is a camera as it is a noninvasive sensor with a reasonable price and copes well with surface conditions whose 2D visual characteristics are unique and obvious. Examples of road surface conditions that are popular among 2D computer vision solutions are cracks as previously studied by [15, 18, 25] and potholes as previously studied by [6, 18, 27].

Although analyzing images for the purpose of road health inspection is not new, since the work of Zhang et al. [31] in 2016, this research area has been dominated significantly by deep learning solutions. Thereby, exploring recent works in image-based road health inspection systems infers exploring why or why not researchers utilized AutoML in their deep learning systems. In contrast to the rising trend of AutoML, to the best of our knowledge, we found no leading research paper in this area utilizing AutoML. To demystify this, we will go through some recent research papers selected based on the proposed year (the newer the better) and different types of basic computer vision tasks (i.e., image segmentation, object detection, and image classification).

#### Image segmentation task

In the work of [26] published in October 2021, although the initial image dataset and output classes were borrowed from other previous work, the authors still had to perform additional crack region mask annotation by themselves. As most AutoML frameworks work in a setting of supervised learning, automate data preparation and annotation are clearly out of AutoML’s scope. In the part of data augmentation where it could be replaced by AutoML, the authors chose three augmentation techniques manually in order to reasonably align their selection with image diversity in real-life situations (e.g., brightness augmentation for different weather conditions, blur augmentation for different camera’s depth of fields, and contrast augmentation for varied illuminations). Then, the three augmentation techniques were superimposed using random combinations. For the part of designing a deep learning model and choosing a set of hyperparameters, the authors seemed to design everything by themselves based on their research hypotheses. As the deep learning model in this work aimed for an image segmentation task that recent AutoML frameworks have not yet supported, AutoML was not applied in this work to no one’s surprise. In addition, a deep learning-based image segmentation task usually involves high GPU memory consumption during model training and this work of [26] utilizes just one Nvidia RTX 2080 Ti GPU (11GB). Therefore, without carefully choosing a model architecture and its hyperparameters, the model will easily exceed the GPU memory consumption, resulting in an out-of-memory error in training.

Back in 2018, we published our work about image-based pothole detection [27] that relied on neither machine learning nor deep learning techniques; all tasks and decisions were carefully designed and fabricated by pure human knowledge. Later in 2021, we published another work of image-based pixel-level road crack detection using deep learning [28]. Comparing our experiences from these two previous works, the latter work in 2021 was superior in terms of accurate results that were more robust to unpredictable visual artifacts of actual road images. However, the first work from 2018 was cheaper in terms of experimental resources as it required just a few image samples, a normal CPU computer, and a not-so-long experimental time. In other words, it was human knowledge that help reduce the requirement for data and other computational resources. This is in contrast to the work from 2021 that assumed a lot of training resources as it was a basic requirement when training a deep learning model. Fortunately, we knew that there existed the U-Net deep learning architecture that could be trained from scratch with a few image samples. By selecting this U-Net architecture based on our knowledge, the number of training samples as well as the number of trial-and-error network architectures were sharply reduced. Despite this resource consumption reduction, the experimental period of our 2021 work was still much longer than that of our 2018 work. This is because it took about 1.5–3 h per one model training using our GPU and there were a lot of experiments to try. Similar to the case of [26] as explained earlier, our 2021 work did not involve AutoML as it was an image segmentation task that was not supported by leading AutoML frameworks. Also, the 8GB GPU memory limitation did not allow us to choose each model component and its hyperparameters at will. With this limited computational resource in place, it was not possible to unleash the full potential of AutoML. Also, in the limited time, it was not possible for us to wait so long for AutoML to randomly try hundreds or thousands of possibilities until finding the best-performing one.

#### Object detection task

Another research example published in April 2021 is the work of [22] that proposed a cleaning robot whose target was to inspect pavement condition and collect pavement garbage (if any). The authors of this work divided the image-based task into two consecutive sub-tasks—pavement segmentation, and pavement defect as well as garbage detection. The first sub-task was done with a deep learning model architecture for image segmentation named SegNet whereas the second sub-task was done by the deep learning-based object detector named YOLO (via the Darkflow framework). Although the part of object detection was already supported by some AutoML frameworks, the authors of this work mentioned nothing about using AutoML; all model selections were done manually.

Likewise, other works [3, 29] presented in December 2020 involved no AutoML in their systems that applied deep learning-based object detectors for road damage detection. Similar to the work of [22] mentioned earlier, the authors of these two works manually chose state-of-the-art deep learning-based object detector models (e.g., Faster R-CNN and YOLO) and finetuned them on their custom image datasets. Minor architecture modifications (e.g., changing the convolutional neural network backbone) and hyperparameter tuning were performed based on the authors’ assumptions and prior knowledge from previously published works. The work of [3] also conducted experiments by themselves to find the most appropriate choice of model ensemble.

These case study researches of object detection share the same development strategy of using well-known state-of-the-art models and finetuning them to their custom image dataset. The whole development pipeline from collecting data to ensembling models is done manually based on human decisions despite the fact that there are many repetitive tasks that can be done by AutoML. From our point of view, this is because the target of these works is not to propose a brand new model architecture with record-breaking performances but to accomplish desired tasks with reasonable and practical performances. As there exists the obviously promising and popular solution of retraining state-of-the-art object detectors on a custom image dataset, there is no point to wait endlessly for several random attempts from AutoML whose final performances cannot be guaranteed. Besides, it is very expensive to train these complicated object detectors from scratch until they can reach the point of high-level scene/object understanding. Therefore, one trial-and-error attempt of AutoML upon this may refer to several hours or days of training (depending on the dataset, model architecture, hyperparameter, and computing unit) which is definitely unproductive for researchers.

#### Image classification task

According to Fig. 3, it can be seen that image classification is a task that is supported by all deep learning-based AutoML frameworks. This is because image classification is the most mature task in image-based deep learning and has been used to internally power other sophisticated image-based tasks for several years. Nevertheless, like other tasks mentioned earlier, we found that recent research papers on image-based road health inspection did not utilize AutoML for their works. For example, the work of Ebenezer et al. [5], published in October 2021, classified an input image into four types of road damages using a majority-vote ensemble model including one self-designed convolutional neural network, one pre-trained AlexNet (transfer learning), and one pre-trained Xception (transfer learning). The authors mentioned that they manually designed their deep learning architecture in order to carefully select compact alternatives that were suitable for their limited training resources. So despite the maturity of image classification in deep learning, it is back to the problem of limited computational resources that makes researchers refrain from using AutoML.

### Discussion and evaluation

From our exploratory research upon the aforementioned works in “AutoML in image-based road health inspection systems” section, it is obvious that AutoML is currently not a mainstream solution for researchers developing image-based road health inspection systems. Despite the high complexity of deep learning models and their infinite combinations of hyperparameters, most works still prefer setting their own research hypotheses, augmenting their own data (following real-life data diversity), designing their own network (following the research hypotheses), tuning hyperparameters, and ensembling their models manually. After thoroughly observing this trend in actual research communities, we conclude five main reasons why researchers do not apply AutoML in developing their image-based road health inspection systems at this moment.

1. Some image-based tasks are not supported by leading AutoML frameworks yet. This is straightforward, particularly for the segmentation task.
2. In order to get a good-performing model from a huge search space of AutoML, it requires a lot of data, GPU computational resources, and waiting time. Using human knowledge can significantly reduce and shortcut this unless we possess unlimited computational resources to unleash the full potential of AutoML. This is the case of Ebenezer et al. [5] mentioned earlier where the authors prefer choosing their own model architectures regardless of the availability and maturity of image classification support in AutoML.
3. Fabricating a whole new model architecture for each custom dataset is expensive and not always necessary. Using existing architectures or state-of-the-art models is a promising solution that consumes fewer resources and provides more expectable performances. This can be implied from the works of [3, 5, 22, 29] that share the same idea of reusing well-known and existing deep learning architectures instead of using AutoML to discover the whole new architecture for the same task but a different dataset.
4. As AutoML aims to automate the whole machine learning pipeline, customizing or dictating the internal processes of AutoML may not be fully allowed which can become frustrated for serious researchers. For example, in the work of [22], although it is possible to let AutoML search for a new model architecture that can solve everything in one step, the authors choose to divide the problem into two consecutive tasks (i.e., pavement segmentation and garbage detection) and solve each of them separately with an existing deep learning model.
5. Using AutoML adds another layer of unexplainability to the resultant model as no one can give reasons why the model architecture is constructed this way. This concern is not directly discussed in related works of image-based road health inspection systems as transparency is less crucial in these systems. However, in other systems like those for healthcare as discussed in Waring et al. [30], transparency has a high impact on mass adoption. This is similar to the conclusion drawn from the work of Drozdal et al. [4], mentioning that it was necessary to include transparency and understandability in order to increase user trust in AutoML.

Nevertheless, in other application areas, there are many recent works that seriously utilized AutoML as the main part of their research. For example, Chai et al. [2] used AutoGBM to automate the tuning of XGBoost and other tree-based gradient boosting models for the detection of driving distraction. Ravindran et al. [23] used two AutoML frameworks, namely AutoGluon-Tabular (AGT) and H2O AutoML, based on daily meteorological data. Anwar [1] used the AutoML framework named AutoGluon to diagnose COVID-19 using binary classification on 3D CT scans. Hayashi et al. [11] chose Google Cloud AutoML Vision to identify aphids of three species and yielded over 96% of correct identification. Note that these applications are not related to image-based road health inspection as focusing by our work.

## Conclusions

This paper studies the readiness of popular AutoML frameworks from the perspective of machine learning practitioners. Our objective is to portray how the rising trend of AutoML will affect the future job responsibilities of human data scientists, researchers, and practitioners. The three most popular AutoML frameworks according to the survey conducted by Kaggle are concluded to summarize features available in current AutoML frameworks. Then, case studies from image-based road health inspection are described, focusing on tasks where a human can possibly be replaced by AutoML. According to these case studies that strongly involve image-based deep learning models, it can be inferred that although the rising demand for AutoML is obvious, AutoML is still nowhere near becoming a mainstream solution for image-based machine learning practitioners and researchers at this moment due to several reasons, particularly the reason of high computational resource consumption.

Nevertheless, as this paper focuses on road health inspection research papers published in academic conferences and journals, it can imply that most observed behaviors and decisions belong to researchers with proper academic and technical backgrounds in image-based deep learning. Therefore, it is not much surprising that these people choose to speed up their research by jump-starting and shortcutting some experiments with their own knowledge, instead of waiting for AutoML to randomly do the experiment from scratch for a long time in an unexplainable manner. In the future, we think it should be interesting to pursue this exploratory research with non-researchers, novice users, and civil engineers who demand this kind of system but possess little to no background in how to create such a deep learning model. The purpose of this future research direction will be to find out which way is more preferable or delivers more final products in practice-let them use AutoML (high computational burden, long waiting time, limited tasks and features), or let them learn to create an image-based deep learning model by themselves (high learning curve, intermediate to advanced programming skills required, a very active research field where new knowledge is proposed and updated all the time).

## Data Availability

All data generated or analysed during this study are included in this published article.

## Abbreviations

AutoML: Automated Machine Learning
CPU: Central Processing Unit
GPU: Graphics Processing Unit
TPU: Tensor Processing Unit
NAS: Neural Architecture Search
API: Application Programming Interface
GUI: Graphical User Interface
CT scan: Computerized Tomography (CT) scan

## Footnotes

1 https://autodl.chalearn.org/.

2 https://cloud.google.com/automl.

3 https://automl.chalearn.org/.
